# Time course of angiopoietin-2 release during experimental human endotoxemia and sepsis

**DOI:** 10.1186/cc7866

**Published:** 2009-05-05

**Authors:** Philipp Kümpers, Matijs van Meurs, Sascha David, Grietje Molema, Johan Bijzet, Alexander Lukasz, Frank Biertz, Hermann Haller, Jan G Zijlstra

**Affiliations:** 1 Department of Nephrology & Hypertension, Hannover Medical School, Carl-Neuberg-Straße 1, 30625 Hannover, Germany; 2 Department of Critical Care, University Medical Center Groningen, Hanzeplein 1, 9713 GZ, Groningen, The Netherlands; 3 Department of Pathology and Medical Biology, University Medical Center Groningen, Hanzeplein 19713 GZ Groningen, The Netherlands; 4 Department of Biometrics, Hannover Medical School, Carl-Neuberg-Straße 1, 30625 Hannover, Germany

## Abstract

**Introduction:**

Endothelial activation leading to vascular barrier breakdown denotes a devastating event in sepsis. Angiopoietin (Ang)-2, a circulating antagonistic ligand of the endothelial specific Tie2 receptor, is rapidly released from Weibel-Palade and has been identified as a non-redundant gatekeeper of endothelial activation. We aimed to study: the time course of Ang-2 release during human experimental endotoxemia; the association of Ang-2 with soluble adhesion molecules and inflammatory cytokines; and the early time course of Ang-2 release during sepsis in critically ill patients.

**Methods:**

In 22 healthy volunteers during a 24-hour period after a single intravenous injection of lipopolysaccharide (LPS; 4 ng/kg) the following measurement were taken by immuno luminometric assay (ILMA), ELISA, and bead-based multiplex technology: circulating Ang-1, Ang-2, soluble Tie2 receptor, the inflammatory molecules TNF-alpha, IL-6, IL-8 and C-reactive protein, and the soluble endothelial adhesion molecules inter-cellular adhesion molecule-1 (ICAM-1), E-selectin, and P-selectin. A single oral dose of placebo or the p38 mitogen activated protein (MAP) kinase inhibitor drug, RWJ-67657, was administered 30 minutes before the endotoxin infusion. In addition, the course of circulating Ang-2 was analyzed in 21 septic patients at intensive care unit (ICU) admission and after 24 and 72 hours, respectively.

**Results:**

During endotoxemia, circulating Ang-2 levels were significantly elevated, reaching peak levels 4.5 hours after LPS infusion. Ang-2 exhibited a kinetic profile similar to early pro-inflammatory cytokines TNF-alpha, IL-6, and IL-8. Ang-2 levels peaked prior to soluble endothelial-specific adhesion molecules. Finally, Ang-2 correlated with TNF-alpha levels (r = 0.61, *P *= 0.003), soluble E-selectin levels (r = 0.64, *P *< 0.002), and the heart rate/mean arterial pressure index (r = 0.75, *P *< 0.0001). In septic patients, Ang-2 increased in non-survivors only, and was significantly higher compared with survivors at baseline, 24 hours, and 72 hours.

**Conclusions:**

LPS is a triggering factor for Ang-2 release in men. Circulating Ang-2 appears in the systemic circulation during experimental human endotoxemia in a distinctive temporal sequence and correlates with TNF-alpha and E-selectin levels. In addition, not only higher baseline Ang-2 concentrations, but also a persistent increase in Ang-2 during the early course identifies septic patients with unfavorable outcome.

## Introduction

Microvascular capillary leakage resulting in tissue edema, vasodilation refractory to vasopressors, and increased recruitment of leukocytes denote key features of sepsis-related endothelial-cell activation. During the course of severe sepsis and septic shock, widespread endothelial cell activation contributes to the initiation and progression of multi-organ failure [1]. Recently, Angiopoietin (Ang)-2 has emerged as a key regulator of endothelial cell activation [2]. In critically ill patients, Ang-2 increases endothelial permeability and is considered a key molecule in the pathogenesis of acute lung injury (ALI) and acute respiratory distress syndrome (ARDS) [3,4].

Ang-1 and Ang-2 are antagonistic ligands, which bind to the extracellular domain of the Tie2 receptor, which is almost exclusively expressed by endothelial cells [5,6]. Binding of the agonist Ang-1 to the endothelial Tie2 receptor maintains vessel integrity, inhibits vascular leakage, suppresses inflammatory gene expression, and prevents recruitment and transmigration of leukocytes [7,8]. In contrast, binding of Ang-2 to the Tie2 receptor disrupts protective Ang-1/Tie2 signaling and facilitates endothelial inflammation in a dose-dependent fashion [9].

*In vitro*, Ang-2 simultaneously mediates disassembly of cell–cell and cell–matrix contacts, and causes active endothelial cell contraction in a Rho kinase-dependent fashion, followed by massive plasma leakage and loss of vasomotor tone [3,10]. Furthermore, Ang-2 facilitates up-regulation of inter-cellular adhesion molecule-1 (ICAM-1), vascular cell adhesion molecule -1 (VCAM-1), and E-selectin [3,7,10,11].

*In vivo*, Ang-2-deficient mice do not exhibit any vascular inflammatory responses in experimental sepsis, and vessels in Ang-1-overexpressing mice are resistant to leakage to inflammatory stimuli [12,13]. As a Weibel-Palade body-stored molecule (WPB), Ang-2 is rapidly released upon endothelial stimulation and is regarded the dynamic regulator within the Ang/Tie system [7,12]. Consistently, exceptionally high levels of circulating Ang-2 have been detected in critically ill patients with sepsis and sepsis-related organ dysfunction [14-16].

Beyond its role as a mediator, Ang-2 has been identified as a promising strong marker of endothelial activation in various diseases [17-19]. In critically ill septic patients, we recently showed that admission levels of circulating Ang-2 correlates with surrogate markers of tissue hypoxia, disease severity, and is a strong and independent predictor of mortality [20]. However, the exact time course of Ang-2 release during sepsis and the role of inflammatory cytokines thereof remain elusive. Furthermore, the tempting sequential concept [7] of Ang-2 as a primer for excess endothelial adhesion molecule (e.g. ICAM-1, VCAM-1, and E-selectin) expression in sepsis has not been investigated in human sepsis.

To address these issues, we wanted to study the time course of Ang-2 release, and the association of Ang-2 with soluble adhesion molecules and inflammatory cytokines in a graded and well-defined human endotoxemia model. Therefore, we re-measured circulating Ang-2, cytokines, and adhesion molecules in blood samples from a placebo-controlled interventional trial on pharmacologic p38 mitogen-activated protein (MAP) kinase inhibition during experimental human endotoxemia [21]. Furthermore, we analyzed circulating Ang-2 in septic patients during a 72 hour time course after admission to the intensive care unit (ICU).

## Materials and methods

### Subjects

Twenty-one healthy male subjects, mean age 29 (range 19 to 44) years, were admitted to the research unit of our ICU (Medical Department) at University Medical Center of Groningen, Groningen, The Netherlands. The local Medical Ethics Committee approved the study and written informed consent was obtained from the subjects. A radial arterial catheter was placed for blood sampling. Thirty minutes before the infusion of lipopolysaccharide (LPS), the volunteers received a single oral dose of RWJ-67657 (4-(4-(Fluorophenyl)-1-(3-phenylpropyl)-5-(4-pyrindinyl)-1H-imidazol-2-yl)-3-butyn-1-ol), supplied in an oral pharmaceutical formulation (R.W. Johnson Pharmaceutical Research Institute, Bassersdorf, Switzerland). Three dose levels were tested, placebo-controlled: placebo (n = 6), 350 mg (n = 5), 700 mg (n = 6), and 1400 mg (n = 4). At time point t = 0, LPS (*Escherichia coli*, batch EC-6, US Pharmacopeia, Twinbrook Parkway, Rockville, MD, USA) was administered as a one minute infusion at a dose of 4 ng/kg body weight (10.000 LPS units/mg). Blood samples were drawn at several time points between pre-medication (t = 0) and 24 hours after administration of LPS. Samples were placed on ice, centrifuged, stored at -80°C, and analyzed in a blinded fashion [21].

### Patients

The time course of Ang-2 release during the early course of human sepsis was studied in 21 ICU patients (Internal Medicine Department) recruited at Hannover Medical School (tertiary care university hospital), Hannover, Germany. Patient characteristics are shown in Table 1. Enrollment was performed after obtaining written informed consent from the patient or his/her legal representatives. If the patient was recovering and able to communicate, he/she was informed of the study purpose and consent was required to further maintain status as a study participant. Twenty-eight day survival was the primary outcome studied and was calculated from the day of ICU admission to day of death from any cause. Patients who did not die within the follow-up were censored at the date of last contact. The study was carried out in accordance with the declaration of Helsinki and was approved by the institutional review board. Serum samples were obtained at baseline (admission), 24 hours, and 72 hours, placed on ice, centrifuged, stored at -80°C, and analyzed in a blinded fashion.

**Table 1:** Characteristics of septic ICU patients on admission

**Characteristics**	**Value**
**Patients**, number	21
Male	8 (38.1%)
Female	13 (61.9%)
**Age**, years, median (min to max)	57 (36 to 72)
**Reason for medical ICU admission**	
Pneumonia	12 (57.1%)
Peritonitis	4 (19.0%)
Urinary tract infection	2 (9.5%)
Systemic mycosis	2 (9.5%)
Endocarditis	1 (4.8%)
Mediastinitis	1 (4.8%)
**Mean arterial pressure **(mmHg)	78 (58 to 108)
**Heart rate **(beats/minute)	95 (53 to 125)
**Vasopressor support**, number	12 (57.1%)
**Mechanically ventilated**, number	19 (90.5%)
Fraction of inspired oxygen (%)	40 (25 to 95)
**APACHE II score**	22 (12 to 48)
**SOFA score**	10 (3 to 19)
**Mortality**, number	11 (52.4%)

APACHE = Acute Physiology and Chronic Health Evaluation; ICU = intensive care unit; SOFA = Sequential Organ Failure Assessment.

### Quantification of circulating angiopoietin-1 and 2, and soluble Tie2

Ang-1 and Ang-2 were measured by in-house immuno luminometric assay (ILMA), and ELISA as previously described [17,18,20]. Soluble Tie2 was measured by commercially available ELISA kit (R&D Systems, Oxford, UK) according to the manufacturer's instructions.

### Quantification of soluble endothelial-adhesion molecules and cytokines

Soluble ICAM-1, E-selectin, and P-selectin were measured using Fluorokine^® ^MultiAnalyte Profiling kits and a Luminex^® ^Bioanalyzer (R&D Systems, Oxford, UK) according to the manufacturers' instructions. TNF-alpha, IL-6, IL-8, and c-reactive protein (CRP) were determined using Medigenix Easia kits from BioSource (BioSource, Nivelles, Belgium) and reported previously [22].

### Statistical analysis

The modified Kolmogorov-Smirnov test was used to test for a normal distribution of continuous variables. In the human endotoxemia model, a non-parametric analysis of variance (ANOVA) (Friedman's test) with Dunn's test for multiple comparison (two-sided) was used to demonstrate statistical changes in Ang-2, cytokines, and adhesion molecules. Correlations of Ang-2 with TNF-alpha, E-selectin, and the heart rate/mean arterial pressure index were calculated with Pearsons's correlation and linear regression analysis after log-transformation. Data are presented as mean ± standard error of the mean unless otherwise stated.

In patients, differences between survivors and non-survivors at baseline and during follow-up were compared by non-parametric two-sided Mann Whitney U test. Receiver operator characteristic (ROC) procedures identified optimal cut-off values for Ang-2 to differentiate between survivors and non-survivors. Contingency table-derived data and likelihood ratios were calculated using the StatPages website [23]. Two-sided *P *< 0.05 were considered statistically significant for all statistical procedures used. All statistical analyses were performed using the SPSS package (SPSS Inc., Chicago, IL, USA) and the GraphPad Prism software (GraphPad Prism Software Inc. San Diego, CA, USA).

## Results

### Angiopoietin-2 is released in a distinctive pattern after endotoxin challenge in healthy volunteers

**Figure 1:**
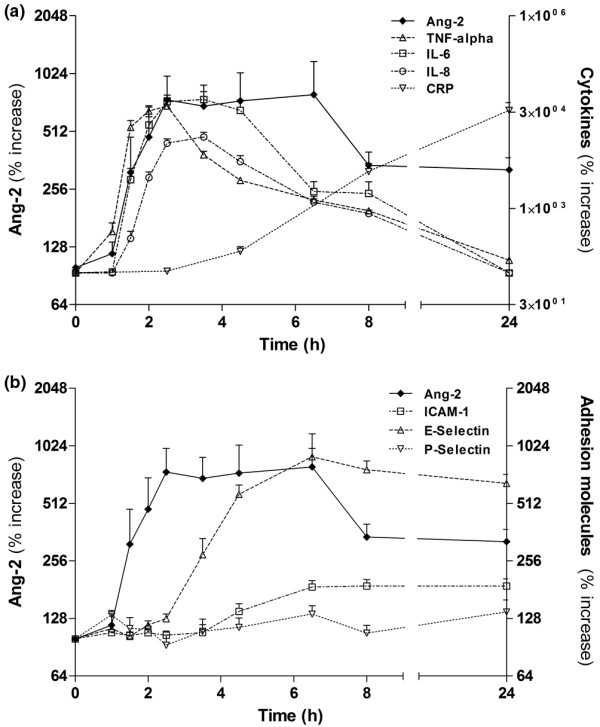
Time course of Ang-2, cytokines, and adhesion molecules after LPS challenge in healthy subjects. **(a) **Concentrations of circulating angiopoietin (Ang)-2 compared with plasma levels of TNF-alpha. IL-6, IL-8, and C-reactive protein (CRP) after lipopolysaccharide (LPS) challenge in six healthy volunteers. **(b) **Concentrations of circulating Ang-2 compared with plasma levels of endothelial adhesion molecules E-selectin, P-selectin, and inter-cellular adhesion molecule-1 (ICAM-1) after LPS challenge in six healthy volunteers. Non-parametric analysis of variance (Friedman's test) with Dunn's test for multiple comparison (two-sided) was used to demonstrate statistical changes in Ang-2, cytokines, and adhesion molecules (y-axes denote percentage increase; baseline = 100%).

Normal Ang-2 concentrations (0.57 ± 0.20 ng/mL) were present at baseline in healthy volunteers (Table 2). Ang-2 levels started to increase at two hours, were significantly elevated from 2.5 hours until 6.5 hours (*P *< 0.01), reaching peak levels (2.42 ± 0.54 ng/mL) 4.5 hours after LPS infusion (*P *< 0.0001; Figure 1; n = 6, placebo group).

**Table 2:** Time course after LPS challenge in healthy subjects

**Time course after LPS challenge**											
**Variables**	**Pre-dose**	**1 hour**	**1.5 hours**	**2 hours**	**2.5 hours**	**3.5 hours**	**4.5 hours**	**6.5 hours**	**8 hours**	**24 hours**	*P *value

**Systolic BP **(mmHg)	140 ± 14	139 ± 11	152 ± 11	158 ± 18	151 ± 18	142 ± 22	121 ± 18	107 ± 11	104 ± 11	131 ± 11	< 0.0001
**Diastolic BP **(mmHg)	74 ± 10	74 ± 7	80 ± 7	77 ± 12	65 ± 12	61 ± 14	54 ± 11	55 ± 8	55 ± 7	66 ± 7	< 0.0001
**MAP **(mmHg)	96 ± 11	96 ± 9	104 ± 7	104 ± 13	94 ± 14	88 ± 16	77 ± 13	72 ± 8	71 ± 7	88 ± 7	< 0.0001
**Heart rate **(beats/minute)	61 ± 16	59 ± 11	78 ± 19	78 ± 18	92 ± 12	98 ± 8	101 ± 8	97 ± 11	96 ± 13	81 ± 16	< 0.0001
**HR/MAP index**	0.64 ± 0.11	0.62 ± 0.11	0.75 ± 0.17	0.76 ± 0.22	1.01 ± 0.22	1.15 ± 0.26	1.36 ± 0.32	1.36 ± 0.27	1.4 ± 0.22	0.92 ± 0.16	< 0.0001
**Body temperature **(°C)	35.4 ± 0.38	36.2 ± 0.54	36.4 ± 0.87	37.0 ± 1.04	37.6 ± 1.11	38.5 ± 0.66	38.9 ± 0.53	38.14 ± 0.28	37.9 ± 0.19	36.2 ± 0.29	< 0.0001
**White blood count **(10^3^/μl)	5.5 ± 0.7	-	-	-	-	-	-	-	-	10.9 ± 1.6	0.03
**C-reactive protein **(mg/l)	1.1 ± 2.4	0.9 ± 1.8	-	-	1.5 ± 2.4	2.2 ± 2.5	1.1 ± 2.3	1.8 ± 2.8	4.8 ± 2.0	60.0 ± 21.5	< 0.0001
**Ang-1 **(ng/ml)	67.0 ± 20.7	58.2 ± 24.4	-	-	61.2 ± 25.0	54.3 ± 19.5	-	64.9 ± 29.1	60.3 ± 31.4	52.3 ± 21.6	0.053
**Ang-2 **(ng/ml)	0.57 ± 0.50	0.63 ± 0.20	1.04 ± 0.65	1.63 ± 0.89	2.33 ± 0.69	2.35 ± 1.06	2.42 ± 1.32	2.23 ± 1.18	1.61 ± 1.07	1.51 ± 1.03	< 0.0001
**Tie2 **(ng/ml)	1.34 ± 0.31	1.23 ± 0.29	1.33 ± 0.32	1.53 ± 0.52	1.23 ± 0.20	1.3 ± 0.16	1.31 ± 0.35	1.4 ± 0.3	1.25 ± 0.32	1.43 ± 0.42	0.085

A non-parametric repeated-measures analysis of variance (Friedman's test) was used to test for significant changes of variables during the time course after LPS challenge (placebo group; n = 6). ANG = angiopoietin; BP = blood pressure; HR = heart rate; LPS = lipopolysaccharide; MAP = mean arterial pressure.

In our cohort of healthy volunteers, neither endogenous sTie2, nor circulating Ang-1 concentrations changed during 24 hours after endotoxin challenge (Table 2).

### Angiopoietin-2 release runs in parallel with early pro-inflammatory cytokines and precedes endothelial inflammation after endotoxin challenge

Plasma levels of TNF-alpha were already significantly elevated at 1.5 hours (*P *< 0.01) compared with baseline, and 30 minutes earlier compared with Ang-2 and IL-6 (Figure 1a). IL-8 appeared in the circulation about 30 minutes later than Ang-2 and IL-6. Elevated Ang-2 levels declined more slowly than that of TNF-alpha, IL-6, and IL-8.

Soluble E-selectin appeared in the circulation later than Ang-2 and E-selectin levels were elevated from 4.5 hours until 24 hours (all *P *< 0.0001). Similarly, ICAM-1 levels were elevated from 6.5 hours until 24 hours after LPS infusion (all *P *< 0.0001; Figure 1b). However, P-selectin did not increase after endotoxin challenge in the present study (*P *= 0.151).

### Angiopoietin-2 release after endotoxin challenge is attenuated by p38 MAP kinase inhibition

Our previous studies have shown that inhibition of the intracellular p38 MAP kinase attenuated inflammatory responses during human endotoxemia [21]. Thus, we hypothesized that p38 MAP kinase inhibition would also have an impact on Ang-2 release. In addition to LPS-treated subjects that received placebo (n = 6), circulating Ang-2 was determined in LPS-treated subjects that were randomized to different doses of an oral p38 MAP kinase inhibitor [21]. In contrast to the placebo group (LPS without p38 MAP kinase inhibitor), no statistically significant Ang-2 release occurred in any of the three interventional groups (i.e. 350 mg, 700 mg, or 1400 mg of RWJ-67657). However, when the areas under the curves (AUC) during the time course were calculated, a dose dependent effect of RWJ-67657 on Ang-2 release was present.

The AUC of absolute Ang-2 values (ng/ml) were 39.8, 31.0, 32.1, and 17.8 in the placebo and the three interventional groups, respectively. Correspondingly, the AUC of percentage increase in Ang-2 from baseline were 9850, 4765, 3435, and 2567 in the placebo and the three interventional groups, respectively.

### Circulating angiopoietin-2 correlates with TNF-alpha levels, soluble E-selectin levels, and the heart rate/mean arterial pressure index

**Figure 2:**
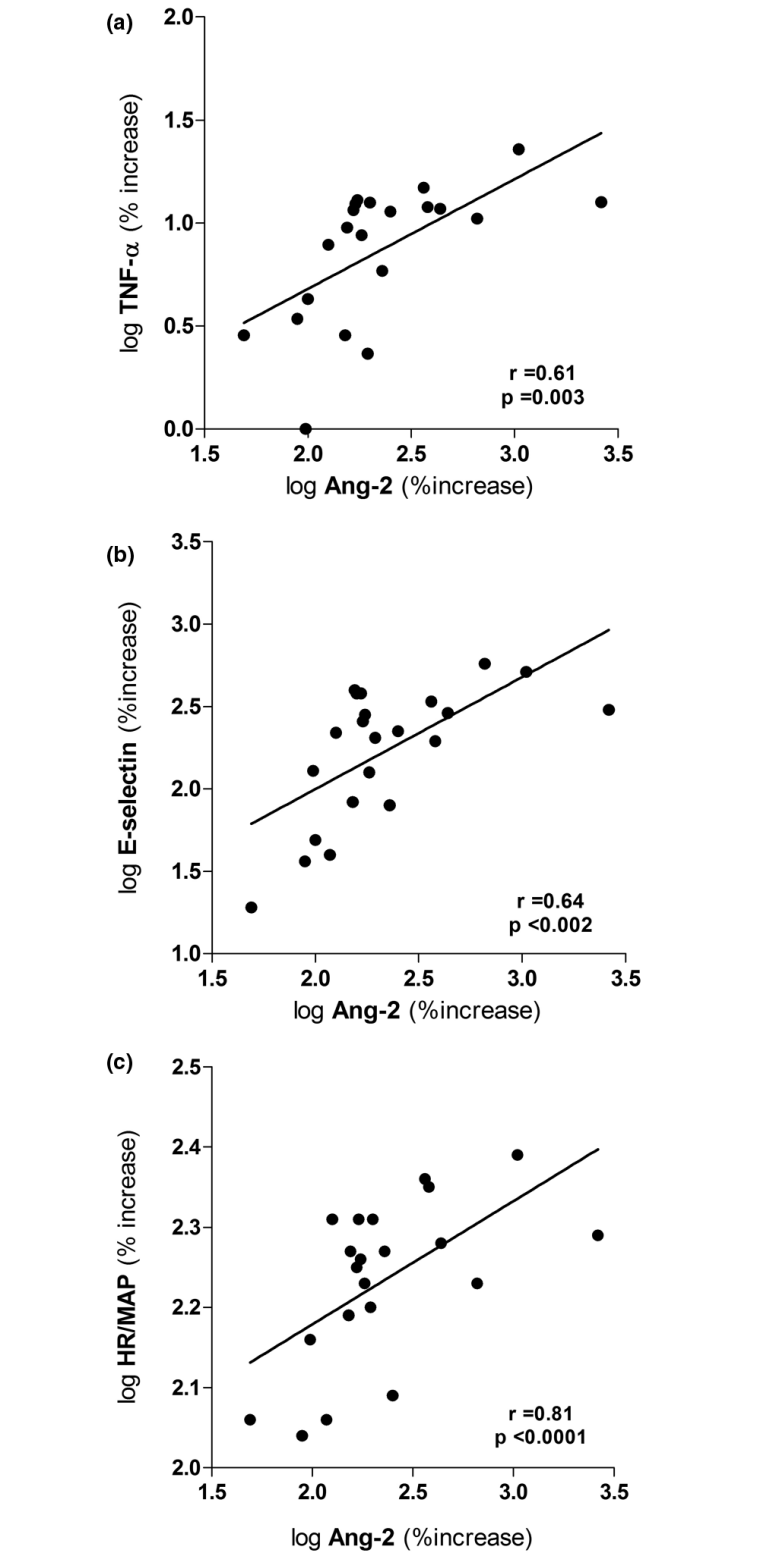
Correlation of Ang-2 with TNF-α, E-selectin and heart rate/mean arterial pressure index after LPS challenge in healthy subjects. Dot blots showing the correlation between circulating angiopoietin (Ang)-2 and **(a) **plasma levels of TNF-alpha, **(b) **plasma levels of the soluble endothelial specific adhesion molecule E-selectin, and **(c) **the heart rate/mean arterial pressure index (HR/MAP index) at 6.5 hours after lipopolysaccharide (LPS) challenge in 21 subjects (placebo (n = 6), and medication groups: 350 mg (n = 5). 700 mg (n = 6), and 1400 mg (n = 4), respectively). Pearsons correlation coefficient was used after logarithmic transformation of variables (axes denote percentage increase after logarithmic transformation; baseline = 100%).

TNF-alpha levels correlated well with Ang-2 at 3.5 (r = 0.44, *P *= 0.04), 4.5 hours (r = 0.54, *P *= 0.012), 6.5 hours (r = 0.61, *P *= 0.003), and 8 hours (r = 0.49, *P *= 0.024; Figure 2a). Likewise, levels of soluble E-selectin were closely associated with Ang-2 at 4.5 hours (r = 0.5, *P *= 0.005), 6.5 hours (r = 0.64, *P *= 0.0013), and 24 hours (r = 0.69, *P *< 0.0004; Figure 2b), when all subjects in the endotoxin model were analyzed (n = 21). Finally, we analyzed the increase in heart rate/mean arterial pressure index as a dynamic surrogate marker of hemodynamic compromise. Indeed, a close correlation was found between the increase in circulating Ang-2 and the increase in heart rate/mean arterial pressure index at 4.5 hours (r = 0.6, *P *= 0.003), 6.5 hours (r = 0.58, *P *= 0.006), and 8 hours (r = 0.75, *P *< 0.0001; Figure 2c), when all subjects were analyzed (n = 21).

### Excess Ang-2 on admission and increasing Ang-2 level during the early course indicate unfavorable 28-day survival in septic patients

**Figure 3:**
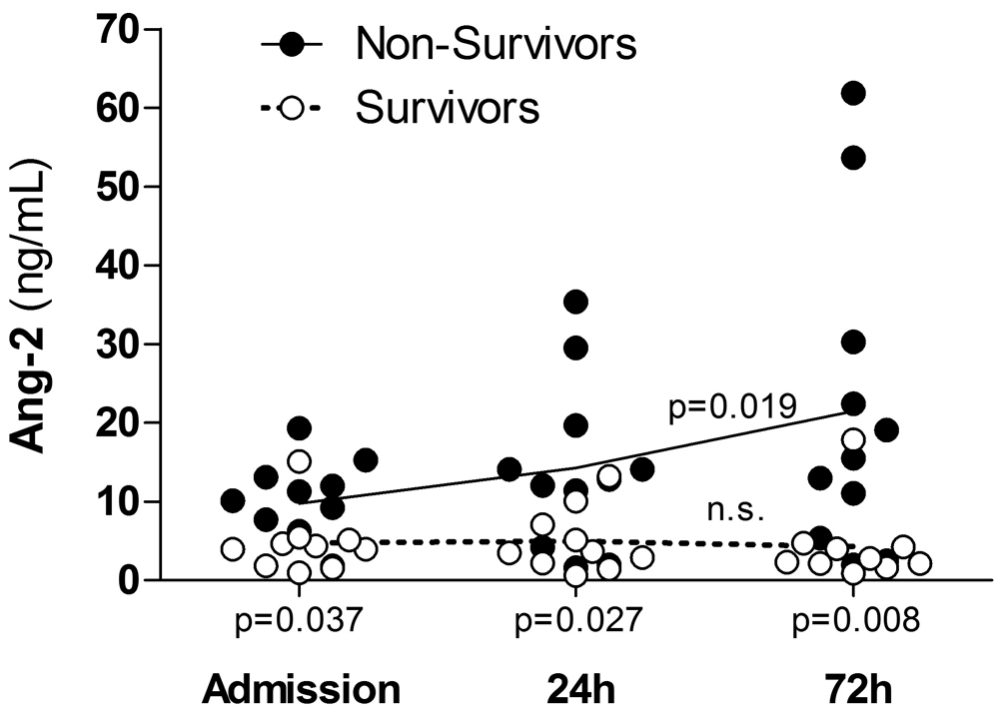
Time course of Ang-2 in critically ill patients with sepsis. Dot blots showing the concentration of angiopoietin (Ang)-2 (ng/ml) in 21 septic on intensive care unit (ICU) admission, 24 hours and 72 hours after admission, respectively. Of note, median Ang-2 levels increased in non-survivors (*P *= 0.019) (continuous line), but remained unchanged in survivors (*P *= 0.83) (dotted line) during the time course. Mean Ang-2 level was higher in non-survivors (filled circles, n = 11) compared with survivors (open circles, n = 10) on admission (*P *= 0.032), after 24 hours (*P *= 0.027), and 72 hours (*P *= 0.008) (two-sided Mann-Whitney test).

First, circulating Ang-2 on admission was 9.8 ± 3.2 ng/ml in septic patients (n = 21). Regarding the kinetics of Ang-2 during follow-up, mean Ang-2 levels remained unchanged at 24 hours (14.3 ± 4.0 ng/ml) and 72 hours (18.2 ± 6.0 ng/ml) when all patients were analyzed (non-parametric repeated measures ANOVA (Friedman's test); *P *= 0.146; Figure 3). Second, when analyzed separately, non-survivors (n = 11) had higher Ang-2 levels compared with survivors (n = 10) on admission (9.7 ± 1.6 ng/ml vs. 4.7 ± 1.3 ng/ml; *P *= 0.032), after 24 hours (13.3 ± 3.2 ng/ml vs. 5.0 ± 1.3 ng/ml; *P *= 0.027) and 72 hours (21.5 ± 6.0 ng/ml vs. 4.3 ± 1.6 ng/ml; *P *= 0.008). In non-survivors, Ang-2 levels were significantly increased after 72 h (9.7 ± 1.6 ng/ml vs. 21.5 ± 6.0 ng/ml; *P *= 0.019). In contrast, no increase in Ang-2 level was detected in survivors during follow-up (4.7 ± 1.3 ng/ml vs. 4.3 ± 1.6 ng/ml; *P *= 0.83; Figure 3).

Finally, we calculated sensitivity, specificity, and predictive values by 2 × 2 tables including all patients (n = 21) to compare the predictive value between absolute Ang-2 at baseline, absolute Ang-2 at 72 hours, and the decrease/increase of Ang-2 between baseline and during 72 hours. At baseline (admission), a ROC-optimized Ang-2 cut-off value more than 5.9 ng/ml best identified non-survivors with 90% specificity and 81% sensitivity. The positive predictive value was 90% and the negative predictive value 81%. In patients with Ang-2 values more than 5.9 ng/ml, the odds ratio (OR) was 40.5 (95% confidence interval (CI) = 3.7 to 398.1) for death during 28-day follow-up (Fisher exact test *P *= 0.002). Essentially the same results were obtained at 72 hours when a ROC-optimized Ang-2 cut-off value of more 5.0 ng/ml was used (Fisher exact test *P *= 0.002). In a similar fashion, albeit with a lower statistical significance, the Ang-2 time course (as a categorical variable: increase vs. non-increase in Ang-2 during 72 hours) identified non-survivors with 81% specificity and 80% sensitivity. The positive predictive value was 81% and the negative predictive value 80%. In patients with increasing Ang-2 values (during 72 hours), the OR was 18.0 (95% CI = 2.2 to 144.6) for death during 28-day follow-up (Fisher exact test *P *= 0.009).

## Discussion

The present study dissects the time course of Ang-2 release after experimental LPS administration in healthy subjects. The decisive results are: LPS (4 ng/ml) is a triggering factor for Ang-2 release *in vivo*; circulating Ang-2 reached peak levels 4.5 hours after LPS infusion; Ang-2 exhibited a kinetic profile similar to that of TNF-alpha, IL-6, and IL-8, and peaked explicitly prior to soluble endothelial-specific adhesion molecules, and correlated with TNF-alpha levels, soluble E-selectin levels, and the heart rate/mean arterial pressure index; in septic patients, not only higher baseline Ang-2 but also a persistent increase in Ang-2 predicts unfavorable 28-day survival.

Clinical studies of pathophysiological changes during sepsis are potentially confounded by the absence of a well-defined onset time of inflammation, by significant co-morbid conditions, as well as by considerable delays from the presumed initiation of inflammation until study inclusion. Animal studies, although indispensable for investigating early and late events during systemic inflammation, are potentially confounded by major inter-species differences in the sensitivity and immune response to various types of inflammatory stimuli [24,25]. As already indicated, the present study is a re-analysis of blood samples from a placebo-controlled interventional trial on pharmacologic p38 MAP kinase inhibition in endotoxemia. The design of this trial enabled us to investigate the time course of Ang-2 release in humans in a highly standardized experimental model with a graded inflammatory response [21,22].

After LPS infusion, peak Ang-2 levels (2.4 ± 0.5 ng/ml) are four-fold lower than Ang-2 levels in critically ill patients at the ICU (9.8 ± 3.2 ng/ml). Emerging data from our group, as well as a recent study by Siner and colleagues [16] suggest the notion that survival is good in critically ill patients with low Ang-2 (< 7 to 8 ng/ml), whereas outcome is explicitly worse above this threshold. Indeed, septic patients with circulating Ang-2 levels below 5.9 and 5.0 ng/ml (admission and 72 hours, respectively) identified patients with good 28-day survival in the present study. Compared with the experimental endotoxemia model (single dose of LPS), the inflammatory stimuli in critical illness are probably more intense, often persistent, and multiple in nature. Thus, a rather low but significant Ang-2 peak level of 2.4 ng/ml (four-fold vs. baseline) during experimental endotoxemia is probably adequate and well in line with the aforementioned data. Although we cannot rule out loss of Ang-2 immunoreactivity due to deep-freeze storage for several years, in our experience this phenomenon is negligible [26].

Ang-2 exhibited a kinetic profile that is similar to the early pro-inflammatory cytokines TNF-alpha, IL-6, and IL-8. In the present study, TNF-alpha level increased somewhat earlier than Ang-2 levels did. As previously shown, the release of TNF-alpha and several other cytokines during human endotoxemia is blocked by p38 MAP kinase inhibition in a dose-dependent manner [22]. In the present study, p38 MAP kinase inhibition blocked Ang-2 release in a similar fashion. In addition, Ang-2 correlated well with TNF-alpha throughout the time course after LPS infusion. This implies that either the Ang-2 release is mediated by TNF-alpha, or that a p38-MAP kinase-dependent upstream signaling pathway controls both TNF-alpha and Ang-2 release. Well in line with this data, Orfanos and colleagues reported a strong relationship of Ang-2 with TNF-alpha in critically ill patients, suggesting that the latter may participate in the regulation of Ang-2 production in sepsis [27]. In contrast, Fiedler and colleagues showed that even high concentrations of TNF-alpha are not sufficient to induce Ang-2 release from human umbilical vein cells *in vitro *[28]. However, we cannot exclude that there is an independent route with a slower signaling pathway, and the fact that TNF-alpha preceded Ang-2 release does not prove causality.

Expression of endothelial adhesion molecules such as E-selectin, VCAM-1, and ICAM-1, are a consistent feature of sepsis [29,30]. As a functional antagonist of Ang-1/Tie2 signaling, Ang-2 promotes up-regulation of endothelial adhesion molecules (i.e. endothelial activation), by sensitizing endothelial cells toward cytokine-induced adhesion molecule expression. Consistently, firm leukocyte adhesion and subsequent transmigration is almost absent during experimental sepsis in Ang-2 deficient animals [3,7,12]. In the present study, Ang-2 levels increased and peaked explicitly prior to soluble endothelial-specific adhesion molecules E-selectin and ICAM-1. Furthermore, soluble E-selectin correlated well with circulating Ang-2 throughout the time course. This temporal sequence is in line with the concept proposed by Fiedler and colleagues [7] that endothelial-cell activation might indeed represent a predominantly Ang-2 driven process *in vivo*.

Although (circulating) Ang-2 has a significant adverse effect on pulmonary vascular barrier properties in sepsis [3,4] its role in extra-pulmonary endothelial activation and systemic loss of barrier function is less well defined. However, Ang-1 increases arteriolar vasoconstriction to phenylephrine in the presence of LPS *in vitro *[31] and preserves blood pressure and cardiac output in septic rats *in vivo *[10]. Indeed, we found a close correlation between circulating Ang-2 and the heart rate/mean arterial pressure as a surrogate marker for hemodynamic compromise in the present study. Although this observation does not prove causality, it is in line with a significant association of circulating Ang-2 levels with MAP, and vasopressor requirement in a large cohort of critically ill patients with acute kidney injury (Kümpers P, Hafer C, David S, Kielstein JT, Hecker H, Lukasz A, Fliser D, Haller H, Faulhaber-Walter R, unpublished data).

Over the past few years, it has been appreciated that multiple components of WPB, such as Ang-2, P-selectin, and IL-8, are co-stored with von Willebrand Factor (vWF), the major constituent of WPBs [7]. It has been shown that storage of Ang-2 and P-selectin in WPB is mutually exclusive. Interestingly, we detected a selective release of the aforementioned WPB stored mediators: Ang-2 is released first, then IL-8, and P-selectin is not released at all. The phenomenon that some components are selectively released from WPB has already been show *in vitro *[32] and deserves further attention in *in vitro *studies of differential regulation of WPB exocytosis.

As a prepackaged constituent of WPB, it is not surprising that Ang-2 levels on admission are increased in response to early endothelial activation in critically ill patients [15,16,20]. In line with this finding, high Ang-2 levels on admission are associated with unfavorable 28-day survival. However, it still remained unanswered whether Ang-2 levels either decline or increase during the course of sepsis, as recently summarized by Giuliano and Wheeler [33].

*In vitro *intracellular Ang-2 pools are rapidly replenished after stimulated depletion with a protein kinase C activator (phorbol 12-myristate 13-acetate) [28]. Furthermore, LPS administration has been shown to increase Ang-2 in a murine sepsis model [34]. Based on available data we hypothesized that levels of circulating Ang-2 might even increase in patients with persistent inflammation and/or clinical deterioration. Indeed, in our cohort of septic patients, non-survivors, not only presented with higher admission Ang-2 levels but also showed a significant increase in Ang-2 during a period of 72 hours. It is tempting to speculate that LPS regulates both, the release of Ang-2 from WPB and the transcriptional induction at the same time. However, additional pre-clinical studies and also clinical studies are urgently needed to clarify this issue.

Our study has several limitations. The human endotoxin model carries a risk of inappropriate extrapolation from experimental findings to the clinical setting. However, this is the only model that renders an opportunity to study the early mechanisms of endothelial activation during a time course in human subjects. Because vWF (as the major constituent of WPBs) was not determined in this study and citrated plasma samples from the original trial were not available for re-evaluation, we cannot exclude that Ang-2 might have derived from endothelial cells exclusively. At least murine macrophages seem to express smaller quantities of Ang-2, but this has not been tested in experimental sepsis [35]. However, the time course of Ang-2 release in the present study was well in line with that of vWF release during endotoxemia [36]. Further, the sample size of the septic cohort was small. Thus, ROC procedures and 2 × 2 tables should be interpreted with caution. However, Fisher's exact test (still appropriate even with small sample size) confirmed the significance of our findings. Finally, elevated circulating Ang-2 is not an exclusive feature of endotoxemia and sepsis, but rather reflects endothelial activation and vascular damage in diseases that share a significant inflammatory endothelial phenotype [17,18,37].

## Conclusions

We could show for the first time that LPS administration is a triggering factor for Ang-2 release in men. Circulating Ang-2 appears in the systemic circulation during experimental human endotoxemia in a distinctive temporal sequence and correlates with TNF-alpha and E-selectin levels. In addition, not only higher baseline Ang-2 concentrations, but also a persistent increase in Ang-2 during the early course identifies septic patients with unfavorable outcomes.

## Key messages

• LPS administration is a triggering factor for Ang-2 release in human endotoxemia.

• Circulating Ang-2 appears in the systemic circulation during experimental human endotoxemia in a distinctive temporal sequence and correlates with TNF-alpha and E-selectin levels.

• High Ang-2 concentrations at baseline, as well as a persistent increase in Ang-2 during the early course identifies septic patients with unfavorable outcome.

## Abbreviations

ALI: acute lung injury; Ang: angiopoietin; ANOVA: analysis of variance; ARDS: acute respiratory distress syndrome; AUC: area under the curve; CI: confidence interval; CRP: C-reactive protein; ELISA: enzyme linked immuno sorbent assay; ICAM-1: inter-cellular adhesion molecule-1; ICU: intensive care unit; IL: interleukin; ILMA: immunoluminometric sandwich assay; LPS: lipopolysaccharide; MAP: mitogen activated protein; OR: odds ratio; ROC: receiver operator characteristics; TNF-alpha: tumor necrosis factor-alpha; VCAM-1: vascular cell adhesion molecule-1; WPB: Weibel-Palade body.

## Competing interests

The authors declare that they have no competing interests.

## Authors' contributions

PK had the initial idea, supervised the project, analyzed the data, prepared the figures and wrote the manuscript. MM supervised the project, analyzed the data, made figures and contributed to the manuscript. SD contributed to the idea, identified patients, participated in analysis of the results and contributed to the manuscript. GM participated in analysis of the results and contributed to the manuscript. JB performed the multiplex assays and reviewed the manuscript. AL identified patients, established and performed the immunoassays and reviewed the manuscript. FB collected and analyzed patient data and reviewed the manuscript. HH supervised the project and reviewed the manuscript. JZ designed and supervised the entotoxemia model, enrolled patients, analyzed the data and contributed to the manuscript. PK and MM contributed equally to the work and are both considered first authors.
